# Microbial communities associated with wet flue gas desulfurization systems

**DOI:** 10.3389/fmicb.2012.00412

**Published:** 2012-11-30

**Authors:** Bryan P. Brown, Shannon R. Brown, John M. Senko

**Affiliations:** ^1^Department of Biology, The University of Akron, AkronOH, USA; ^2^Babcock & Wilcox Power Generation Group, Inc. BarbertonOH, USA; ^3^Department of Geology and Environmental Science, The University of Akron, AkronOH, USA

**Keywords:** flue gas desulfurization, thermophiles, microbially influenced corrosion

## Abstract

Flue gas desulfurization (FGD) systems are employed to remove SO_*x*_ gasses that are produced by the combustion of coal for electric power generation, and consequently limit acid rain associated with these activities. Wet FGDs represent a physicochemically extreme environment due to the high operating temperatures and total dissolved solids (TDS) of fluids in the interior of the FGD units. Despite the potential importance of microbial activities in the performance and operation of FGD systems, the microbial communities associated with them have not been evaluated. Microbial communities associated with distinct process points of FGD systems at several coal-fired electricity generation facilities were evaluated using culture-dependent and -independent approaches. Due to the high solute concentrations and temperatures in the FGD absorber units, culturable halothermophilic/tolerant bacteria were more abundant in samples collected from within the absorber units than in samples collected from the makeup waters that are used to replenish fluids inside the absorber units. Evaluation of bacterial 16S rRNA genes recovered from scale deposits on the walls of absorber units revealed that the microbial communities associated with these deposits are primarily composed of thermophilic bacterial lineages. These findings suggest that unique microbial communities develop in FGD systems in response to physicochemical characteristics of the different process points within the systems. The activities of the thermophilic microbial communities that develop within scale deposits could play a role in the corrosion of steel structures in FGD systems.

## Introduction

**Figure 1:**
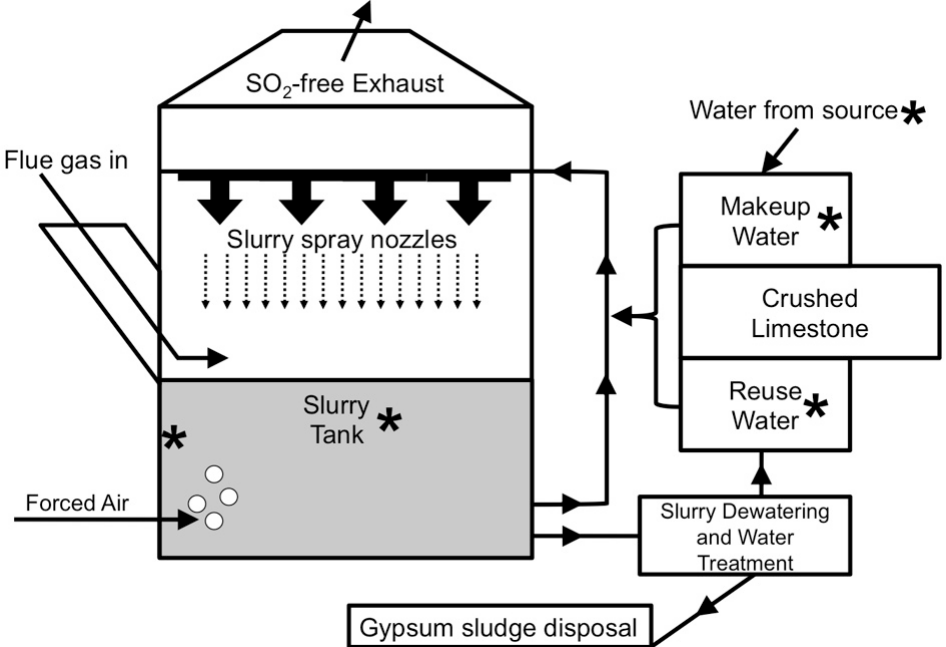
**Schematic depiction of the operation of a limestone-based wet FGD system [adapted from Kiil et al. (1998)].** Asterisks are used to point out locations where samples were obtained from the FGD systems evaluated in this study.

Sulfur-oxide gasses (mostly SO_2_) that are released during the combustion of fossil fuels (most notably from coal-fired electricity generation) are known to cause acid rain (Cullis and Hirschler, 1980). To limit acid rain, flue gas desulfurization (FGD) systems (often referred to as “scrubbers”) are routinely used to remove SO_2_ from the exhaust of coal-fired electric power facilities (Pandy et al., 2005). For limestone-based wet FGD, coal combustion exhaust (flue gas) is diverted into absorber units and passed under a spray of crushed limestone-water slurry (Figure 1), whereupon, flue gas-associated SO_2_ dissolves in the water as sulfite (Equation 1).

(1) $${\text{SO}}_{2}+{\text{H}}_{2}\text{O}\to {\text{SO}}_{3}^{2-}+\text{2 }{\text{H}}^{+}$$

Slurries are initially prepared by mixing the crushed limestone with water sourced from a large body of freshwater (e.g., a lake or river). Slurry spray is collected at the bottom of the absorber unit, and sulfite subsequently precipitates with Ca^2+^ (Equation 2) produced from dissolution of CaCO_3_ in the limestone.

(2) $${\text{Ca}}^{2+}+{\text{SO}}_{3}^{2-}\to {\text{CaSO}}_{3}$$

The O_2_ in forced air that is injected into the absorber unit (Figure 1) facilitates the oxidation of the sulfite moiety of CaSO_3_ to sulfate, forming gypsum (Equation 3).

(3) $$\text{2 }{\text{CaSO}}_{3}+\;{\text{O}}_{2}+\text{ 4 }{\text{H}}_{2}\text{O}\to \text{2 }{\text{CaSO}}_{4}\cdot {\text{2H}}_{2}{\text{O}}_{\text{gypsum}}$$

The slurry at the bottom of the absorber unit may be recirculated through the spray nozzles or subjected to dewatering (to recover gypsum) and water purification (to remove dissolved solids) (Figure 1; Rajendran et al., 1999).

The treated slurry water may be released into a water body or held onsite (as “reuse water”), mixed with fresh limestone, and circulated back into the absorber unit (Figure 1). Slurry water that is lost due to evaporation and during gypsum recovery may be replaced using fresh limestone mixed with makeup water (water obtained from a nearby water body and held in onsite tanks) or reuse water (Figure 1). The interior of FGD absorber units may be considered a physicochemically extreme environment, since (1) heat from the flue gas maintains temperatures of 50–80°C, and (2) absorber unit fluids may contain total dissolved solids (TDS) concentrations of nearly 40 g/l, including approximately 0.5 M Cl^−^ and similarly high SO_4_^2−^ concentrations (Kiil et al., 1998; Nygaard et al., 2004; Kitto and Stultz, 2005; Cooper and Ruocco, 2010; Van Ginkel et al., 2011). High TDS in absorber unit fluids are a result of chemical inputs from coal combustion products, limestone, and source water, as well as the subsequent concentration of dissolved species resulting from evaporation from the absorber unit (Kitto and Stultz, 2005).

Given the abilities of microorganisms to modulate the oxidation states of sulfur species, such activities may be exploited to enhance the performance of FGD systems. For instance, sulfate reducing, Fe(II)- and sulfide-oxidizing, and phototrophic bacterial activities may be exploited for removal of SO_2_ (Dasu et al., 1993; Huber and Stetter, 1998; Pandy et al., 2005; Parshina et al., 2005, 2010), though these activities have not been considered in the context of existing FGD systems. Given the high operating temperatures associated with FGD units, thermophilic microorganisms would be best suited for biotechnological approaches to FGD and SO_2_ removal (Huber and Stetter, 1998). Furthermore, the high TDS of slurry fluids will favor the activities of organisms that are capable of tolerating high osmotic strength fluids. Due to the required oxic conditions, relatively high operating temperatures, high Cl^−^, and SO_4_^2−^ concentration, and potentially low pH of FGD absorber slurries, they represent an extremely corrosive environment (Mansfield and Jeanjaquet, 1987; Hibner and Ross, 1988; Bordziłowski and Darowicki, 1998; Rajendran et al., 1999; Kim et al., 2010). As such, degradation of the steel walls and other structures is a significant concern for FGD operators. More recently, microbially influenced corrosion (MIC) has been proposed to be responsible for the deterioration of the steel walls and other structures associated with absorber units (Moskal, 2011).

Despite the potentially beneficial (i.e., enhanced SO_2_ removal) and detrimental (i.e., MIC) impacts of microbial activities in FGD systems, we are not aware of any studies of the microbiology of operating FGD units. In this study we evaluated the microbial communities associated with the fluids from the wet FGD systems of five coal-fired electric power generation facilities. Samples were collected from various process points, including source water, makeup, and reuse waters, and from slurries inside FGD absorber units (Figure 1). We hypothesized that the physicochemical differences among these various “process points” of the FGD systems would give rise to distinct microbial communities within the broader FGD system. These studies also included the characterization of microbial communities associated with scale deposits recovered from the steel walls of a severely corroded absorber unit and a unit that is not currently exhibiting signs of corrosion.

## Materials and methods

### Facilities, FGD units, and sampling methods

Samples were collected from wet FGD systems of five coal-fired electric power facilities. Samples with their corresponding sample designations are included in Tables 1, 2. Three distinct process points within the FGD systems were sampled, including absorber slurries of Facilities A–E and makeup waters of Facilities A–D. Samples were collected from absorber slurries of two different FGD units at Facilities A–C. Scale deposits on the walls of absorbers at Facilities A and B were collected when these units were drained for periodic maintenance. Slurry samples from Unit 2 of Facilities A and B were collected approximately 1 month after maintenance activities and subsequent startup. Additionally, a sample was collected near the water intake for Facility A from a freshwater body that provides the source water for Facilities A and B. Source waters for Facilities C, D, and E were not sampled for this study. Raw makeup/reuse water, absorber slurry, and source water were collected by filling sterile bottles with operating process fluid by holding them under a flowing stream at the unit sampling port. Hard, mineralized scale deposits from the walls of absorber units that were likely derived from slurry solids were collected by scraping and transferring the deposits to sterile centrifuge tubes with a sterile plastic spatula. All samples were refrigerated and shipped via overnight courier to The University of Akron, where they were processed further. Subsamples for culture-dependent microbial enumerations and evaluation of solid-phase chemistry were stored at 4°C (for no more than 2 week) before further processing (described below). Subsamples for analysis of aqueous chemistry were immediately filter sterilized (0.2 μm pore size) upon receipt in the laboratory and stored at 4°C prior to analysis. Subsamples for nucleic acid-based microbial community analysis were transferred to a −80°C freezer before further processing (described below).

**Table 1:** **Aqueous chemical composition of source, makeup, reuse, and slurry fluids**.

**Sample**	**Sample designation**	**pH**	**Cl^−^ (mM)**	**NO_3_^−^ (mM)**	**SO_4_^2^− (mM)**	**Fe(II) (μM)**	**Mn (mM)**	**Ca (mM)**	**DOC (mg/l)**	**Carbonate Alkalinity (meq/l)**
Facility A Unit 1 absorber slurry	A-U1_slurry_	5.8	413	45	563	n/d	0.96	6.0	118	4.1
Facility A Unit 2 absorber slurry	A-U2_slurry_	6.5	83	15	143	n/d	1.14	16.3	62	7.9
Facility A Unit 1 makeup water	A-U1_makeup_	6.5	0.88	n/d	0.25	n/d	0.55	6.0	9.5	n/a
Facility A source water	A_source_	6.5	1.13	n/d	0.38	n/d	0.64	3.2	4.4	n/a
Facility B Unit 1 absorber slurry	B-U1_slurry_	6.7	453	1	125	n/d	0.05	1.2	64	4.5
Facility B Unit 2 absorber slurry	B-U2_slurry_	6.8	268	n/d	60	n/d	1.21	31.4	28	6.0
Facility B Unit 1 reuse water	B-U1_reuse_	6.6	0.75	n/d	0.63	n/d	0.04	1.6	13	n/a
Facility C Unit 1 absorber slurry	C-U1_slurry_	6.6	335	n/d	760	30	1.83	9.8	179	10.4
Facility C Unit 2 absorber slurry	C-U2_slurry_	6.2	754	n/d	1623	500	0.97	4.7	191	13.5
Facility C Unit 1 makeup water	C-U1_makeup_	6.5	n/d	n/d	n/d	49	n/d	0.6	7.9	n/a
Facility C Unit 2 makeup water	C-U2_makeup_	7.3	n/d	n/d	n/d	55	n/d	0.6	9.3	n/a
Facility D Unit 1 absorber slurry	D-U1_slurry_	6.8	73	10	15	n/d	n/d	16.9	6.0	3.5
Facility D Unit 1 makeup water	D-U1_makeup_	n/a	n/a	n/a	n/a	n/a	n/a	n/a	n/a	n/a
Facility E Unit 1 absorber slurry	E-U1_slurry_	6.7	292	8	115	n/d	0.50	16.9	25	7.1

n/a, not analyzed; n/d, not detected.

**Table 2:** **Solid-phase chemistry of suspended solids in slurry and wall scales from FGD absorber units**.

**Sample**	**Sample designation**	**HCl-extractable Fe(II) (μmol/g)**	**Hydroxylamine-HCl-extractable Fe(III) (μmol/g)**	**HCl-extractable Mn (μmol/g)**	**Hydroxylamine-HCl^−^extractable Mn (μmol/g)**	**[HNO_3_]-extractable Mn (μmol/g)**	**[HNO_3_]-extractable Ca (mmol/g)**
Facility A Unit 1 absorber slurry	A-U1_slurry_	5	36	6	4	11	0.62
Facility A Unit 2 absorber slurry	A-U2_slurry_	3	139	8	8	18	2.70
Facility A Unit 2 wall scale	A-U2_scale_	4	21	7	7	34	5.50
Facility B Unit 1 absorber slurry	B-U1_slurry_	4	20	2	2	5	0.36
Facility B Unit 2 absorber slurry	B-U2_slurry_	34	87	5	5	10	1.60
Facility B Unit 1 wall scale	B-U2_scale_	7	75	2	2	6	5.60
Facility C Unit 1 absorber slurry	C-U1_slurry_	19	64	18	24	45	1.11
Facility C Unit 2 absorber slurry	C-U2_slurry_	21	66	23	22	53	0.53
Facility D Unit 1 absorber slurry	D-U1_slurry_	11	51	2	2	5	2.78
Facility E Unit 1 absorber slurry	E-U1_slurry_	13	51	17	18	35	0.74

### Analytical methods

The pH of source water, makeup waters, and absorber slurries was measured immediately upon receipt in the laboratory. Dissolved anions (Cl^−^, NO_3_^−^, and SO_4_^2−^) in filtered source water, makeup waters, and absorber slurries were quantified by ion chromatography using a Dionex DX-120 ion chromatography system fitted with an AS4 column and conductivity detector (Sunnyvale, CA). Carbonate alkalinity was determined by titration of the fluids with H_2_SO_4_ to a pH of 4.5 (Greenberg et al., 1981). Filtered subsamples intended for analysis of dissolved cations were preserved in 0.5 M HCl, and subsequently analyzed for Fe^2+^ by ferrozine assay (Stookey, 1970) and dissolved Ca and Mn by atomic absorption spectrometry (AA), using a Perkin Elmer Analyst 700 (Waltham, MA). Solid-phases that were suspended in slurries or associated with scale deposits were digested using 0.5 M HCl [operationally defined as solid-associated Fe(II) Lovley and Phillips (1987) and Mn(II/III) Sutter et al. (1974)], 0.25 M hydroxylamine-HCl in 0.25 M HCl [operationally defined as poorly crystalline Fe(III) Lovley and Phillips (1987) and Mn(III/IV) Sutter et al. (1974)], and concentrated HNO_3_ (operationally defined as total Mn and Ca), and remaining solids were removed from suspensions by centrifugation. Extracted Fe(II) was quantified by ferrozine assay (Stookey, 1970), and extracted Mn and Ca were quantified by AA, as described above. Solid contents of absorber slurries were determined gravimetrically after drying 5 ml of slurry.

### Culture-dependent microbial enumerations

Total aerobic organoheterotrophic microorganisms were enumerated using culture-dependent approaches with modified liquid versions of K and Lept media described by Templeton et al. (2005) in a most probable number (MPN) format (Colwell, 1979). Modified Lept medium was buffered with piperazine-N,N′-bis(2-ethanesulfonic acid) (PIPES; pH 6.5) and contained Na_2_SO_4_ (250 mM), glucose (5 mM), MgSO_4_ (0.8 mM), CaCl_2_ (0.8 mM), yeast extract (0.5 g/l), casamino acids (0.5 g/l), and trace metals described by Tanner (1997). K medium was buffered with PIPES (pH 6.5) and contained Na_2_SO_4_ (250 mM), peptone (2 g/l), and yeast extract (0.5 g/l). MPN cultures were incubated aerobically at 50°C. MPN dilution series were scored for heterotrophic growth based on the appearance of turbidity in the medium.

### Nucleic acid-based microbial community characterization

In preparation for extraction of genomic DNA, solids that were suspended in slurry samples were concentrated by centrifugation and the supernatant was removed. Since the slurry solids retained a large volume of water, the remaining solids were lyophilized to maximize the amount of solids from which DNA could be extracted. DNA was subsequently extracted from slurry solid-associated microorganisms (~0.5 g of slurry solids) using MoBio PowerBiofilm DNA isolation kits (MoBio Laboratories, Inc., Carlsbad, CA). Planktonic microorganisms associated with makeup waters or source water was immobilized on hydrophilic polyethersulfone membranes (0.2 μm pore size) by vacuum filtration. Approximately 0.5 l of water was filtered, and approximately half of the 17 cm^2^ filter was subjected to genomic DNA extraction using MoBio PowerBiofilm DNA isolation kits. DNA was quantified using a Nanodrop spectrophotometer (Thermo Scientific Inc., Waltham, MA) and yields ranged from 310 to 1800 ng DNA/sample. Partial sequences of bacterial 16S rRNA genes were obtained using tag-encoded FLX amplicon pyrosequencing at Research and Testing Laboratories, Inc. (Lubbock, TX) as described by Dowd et al. (2008) using Gray28f (5′-TTTGATCNTGGCTCAG-3′) and Gray519r (5′GTNTTACNGCGGCKGCTG-3′) primers. Initial generation of the sequencing library utilized a one-step PCR with a total of 30 cycles, a mixture of HotStart and HotStar high fidelity taq polymerases, and amplicons originating and extending from the 28F position. Tag-encoded FLX amplicon pyrosequencing was conducted using a Roche 454 FLX pyrosequencer with Titanium reagents (Roche 454 Life Sciences, Branford, CT). Following sequencing, all failed sequence reads, low quality sequence ends, and tags and primers were removed. Non-bacterial ribosomal sequences and chimeras were removed using B2C2 (Gontcharova et al., 2010). Short reads (<150 bp), sequences with ambiguous base calls, and sequences with homopolymers >6 bp were removed from the library. Nucleotide sequence libraries obtained in this work have been submitted to the Sequence Read Archive (SRA) under run accession numbers SRR609278 (A_source_), SRR609277 (A-U1_makeup_), SRR609201 (A-U1_slurry_), SRR609275 (A-U2_slurry_), SRR609276 (A-U2_scale_), SRR609281 (B-U1_reuse_), SRR609279 (B-U1_slurry_), SRR609280 (B-U2_slurry_), SRR609282 (B-U2_scale_), SRR609285 (C-U1_makeup_), SRR609283 (C-U1_slurry_), SRR609286 (C-U2_makeup_), SRR609284 (C-U2_slurry_), SRR609288 (D-U1_makeup_), SRR609287 (D-U1_slurry_), and SRR609292 (E-U1_slurry_).

To evaluate the diversity of the various FGD-associated systems, standard rarefaction curves (based on 97% sequence identity) were produced for each unique FGD environment (i.e., absorber scale, absorber slurry, makeup water, and source water) using the Ribosomal Database Project's (RDPs)-II Pyrosequencing Pipeline (Cole et al., 2009). RDP-II Pyrosequencing Pipeline was also used to calculate Shannon and Chao1 diversity indices at a cutoff of 97% sequence identity. Sequences were processed and analyzed further using the MacQIIME (http://www.wernerlab.org/software/macqiime) version of the QIIME software package using default parameters (Caporaso et al., 2010b). Sequences were separated into operational taxonomic units based on 97% sequence identity, and representative OTUs were picked using scripts in the QIIME environment (Edgar, 2010). Taxonomic assignments were made to OTUs using the RDP II's classifier function (Wang et al., 2007) in the QIIME environment. OTU sequences were aligned using the PyNAST algorithm (Caporaso et al., 2010a) against the Greengenes core set (DeSantis et al., 2006), filtered to remove gaps, and a phylogenetic tree was constructed using QIIME. In preparation for beta-diversity analyses, the OTU table from each sample was iteratively rarefied to 1009 sequences using Jack-knife sampling in QIIME. A distance matrix was produced using the weighted UniFrac beta-diversity metric (Lozupone and Knight, 2005), and microbial communities associated with the samples were compared visually using Principal Coordinate Analysis (PCoA) (Lozupone and Knight, 2005).

## Results

### Chemical characteristics of FGD fluids and solids

A_source_, makeup, and reuse waters (referred to collectively as “fresh” waters) contained dissolved chemical species at similar concentrations (Table 1). B-U1_reuse_ contained the highest concentrations of Cl^−^ and SO_4_^2−^ (Table 1), which is likely attributable to the incomplete removal of these dissolved species by slurry water treatment. Slurry fluids contained higher concentrations of dissolved Ca than source waters and carbonate alkalinities ranging from 3.5 to 13.5 meq/l due to limestone dissolution (Table 1). Cl^−^, NO_3_^−^, SO_4_^2−^ concentrations in slurry fluids ranged from 73 to 754 mM, undetectable-45 mM, and 15–1623 mM, respectively (Table 1). WEB-PHREEQ-based geochemical modeling (Saini-Eidukat and Yahin, 1999) using the values contained in Table 1 suggested that all slurry fluids except B-U1_slurry_ were supersaturated with respect to gypsum (CaSO_4_·2H_2_O), and all slurry fluids except A-U1_slurry_, B-U1_slurry_, and E-U1_slurry_ were supersaturated with respect to anhydrite (CaSO_4_). Slurry fluids were enriched in Mn in comparison to source, makeup, or reuse waters, though dissolved Fe(II) concentrations were generally low due to the abundance of oxygen in the fluids (Table 1). Absorber slurries contained considerably higher levels of DOC than “fresh” waters (Table 1), which is likely attributable to non- or partially combusted coal and ash that were not removed by the electrostatic precipitator and entered the absorber unit with the flue gas. HCl-extractable Fe(II) and Mn(II/III) contents of solids ranged from 20 to 34 μmol/g and 1.7 to 23 μmol/g, respectively (Table 2). Hydroxylamine-HCl-extractable Fe(III) and Mn contents of solids ranged from 20 to 139 μmol/g and 1.7 to 24.5 μmol/g, respectively (Table 2). Total Mn and Ca contents ranged from 4.7 to 52.8 μmol/g and 0.36 to 5.6 mmol/g, respectively (Table 2). The variability in concentrations of both dissolved and solid-phase chemical constituents is likely a reflection of variability in raw materials (e.g., water, coal, and limestone) used in the facilities. Electric power generation stations use water from nearby water supplies and frequently utilize locally sourced limestone. Differences in chemical composition of raw materials will in turn influence the chemical composition of absorber fluids. Facilities A and B are ~80 km apart, are owned by the same utility company, and use coal and limestone types, and source waters from the same large freshwater body. Facilities C, D, and E are each located more than 300 km apart, and use different source waters and limestone types.

### Culture-dependent microbial enumerations

The chemical conditions in the absorber unit slurries represent an organic carbon-rich and osmotically challenging environment that is dramatically different from that of the “fresh” waters. Microorganisms are generally classified as halophilic/tolerant based on their ability to grow at high NaCl concentrations (Trüper and Galinski, 1986; Ventosa et al., 1998), and while Cl^−^ concentrations in FGD slurry fluids were quite high, SO_4_^2−^ was generally the dominant anionic constituent (Table 1). As such, we enumerated organotrophic microorganisms in all points of the FGD systems using two types of media that were incubated at 50°C and contained 250 mM Na_2_SO_4_ to specifically target organisms that were able to grow under the osmotic stress imposed by the high sulfate concentrations of the FGD slurry fluids. The physicochemical differences between “fresh” waters and the absorber slurries were reflected in the abundances of organotrophic bacteria detected. When cultured on modified K and Lept media, relatively few halothermophilic/tolerant microorganisms were detected in “fresh” waters in comparison to absorber slurries (Table 3), suggesting that the high temperature and solute concentrations of the absorber units enrich for halothermophilic/tolerant microorganisms. It is notable that of the “fresh” waters, B-U1_reuse_ contained the highest number of halothermophilic/tolerant organisms (Table 3). Since B-U1_reuse_ water is recycled absorber fluid, halothermophilic/tolerant microorganisms were retained in these fluids. While A-U2_scale_ was enriched in halothermophilic/tolerant microorganisms in comparison to A-U1_slurry_ and A-U2_slurry_, B-U2_scale_ contained comparable numbers of halothermophilic/tolerant organoheterotrophs to B-U1_slurry_ and B-U2_slurry_ (Table 3), which may also be attributable to the recirculation of halothermophilic/tolerant organisms in Facility B reuse water (Figure 1).

**Table 3:** **Microbial abundances and diversity estimates from FGD systems**.

**Sample**	Culturable organotrophs (MPN^*^)		**No. of sequences**	**OTU_0.03_ OTUs (%)**	**Genus level-assignable**	**Diversity indices**	
	**Modified Lept medium**	**Modified K medium**				**Shannon**	**Chao1**
A-U1_slurry_	1.1 × 10^7^	9.3 × 10^5^	11,908	184	86	2.3	113
A-U2_scale_	1.2 × 10^8^	5.2 × 10^8^	4954	236	22	3.09	205
A-U2_slurry_	1.5 × 10^3^	4.4 × 10^5^	6009	191	59	2.7	132
A-U1_makeup_	3.5 × 10^2^	3.5 × 10^2^	9381	1865	41	5.9	1628
A_source_	n/d	n/d	1009	335	51	4.1	271
B-U1_slurry_	2.1 × 10^6^	2.4 × 10^7^	5516	113	79	1.8	57
B-U2_scale_	7.6 × 10^7^	1.2 × 10^5^	9271	292	20	2.46	213
B-U2_slurry_	1.5 × 10^5^	4.4 × 10^5^	9634	284	79	3	200
B-U1_reuse_	2.4 × 10^3^	2.4 × 10^3^	6708	330	48	3.5	294
C-U1_slurry_	4.3 × 10^5^	9.3 × 10^5^	8016	163	75	2.4	88
C-U2_slurry_	7.5 × 10^2^	9.3 × 10^2^	4133	110	65	2.8	128
C-U1_makeup_	7.5 × 10^0^	n/d	8319	2260	57	6.3	1956
C-U2_makeup_	2.3 × 10^0^	2.3 × 10^0^	7219	1692	41	5.5	1506
D-U1_slurry_	2.3 × 10^1^	2.1 × 10^3^	5355	123	61	2.4	92
D-U1_makeup_	n/a	n/a	3721	1047	41	5.6	870
E-U1_slurry_	2.1 × 10^6^	1.1 × 10^7^	5437	218	42	2.9	177

* Values represent MPN/g of scale and MPN/ml of water or slurry.

n/a, not analyzed; n/d, not detected.

### Nucleic acid-based community profiling

**Figure 2:**
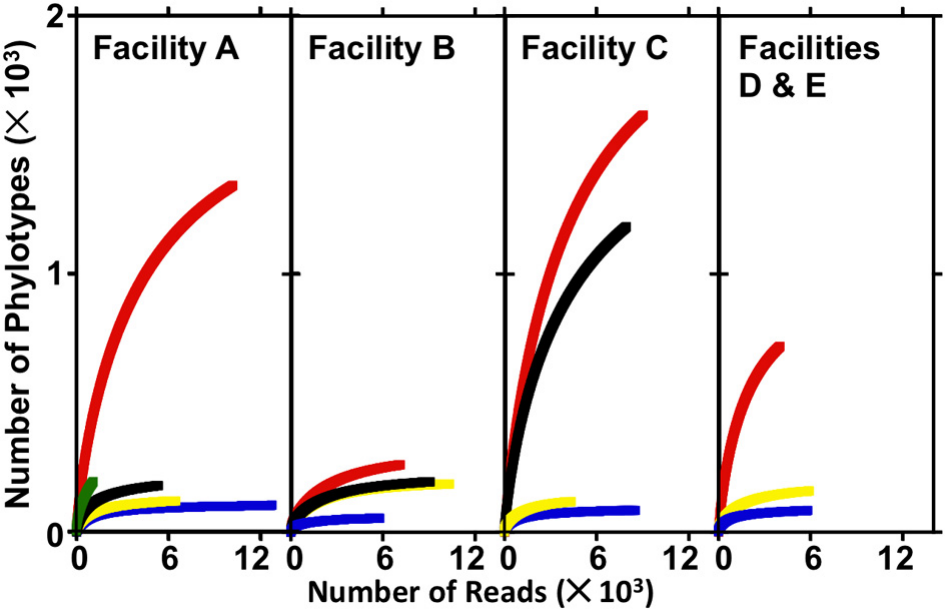
**Rarefaction curves produced from sequence libraries derived from source water, makeup waters, absorber slurries, and absorber scale deposits from electric power facilities A–E.** In facility A, A_source_, A-U1_makeup_, A-U1_slurry_, A-U2_slurry_, and A-U2s_cale_ are depicted by green, red, blue, yellow, and black lines, respectively. In facility B, B-U1_makeup_, B-U1_slurry_, B-U2_slurry_, and B-U2_scale_ are depicted by red, blue, yellow, and black lines, respectively. In facility C, C-U1_makeup_, C-U2_makeup_, C-U1_slurry_, and C-U2_slurry_ are depicted by red, black, blue, and yellow lines, respectively. In facilities D and E, D-U1_makeup_, D-U1_slurry_, and E-U1_slurry_ are depicted by red, blue, and yellow lines, respectively.

The numbers of sequences analyzed, numbers of OTUs, and diversity indices associated with bacterial communities in FGD systems are shown in Table 3. A total of 1,05,769 sequences were analyzed from 16 samples, and average read length was 360 bp. Rarefaction analysis was used to compare the richness of bacterial communities associated with various process points of the FGD systems (Figure 2). While absorber slurries, wall scales, and B-U1_reuse_ were sampled to saturation, source, and makeup waters were not (Figure 2). The highest numbers of OTUs were recovered in the four makeup waters sampled (Table 3), and while only 1009 sequences were obtained from A_source_, this sample exhibited a similar rarefaction pattern to the makeup waters (Figure 2). Non-parametric indicators of diversity showed similar trends, with the highest Chao1 and Shannon richness estimates observed in the makeup waters (Table 3). These results suggest that more complex microbial communities are associated with the makeup and source waters than the more physicochemically extreme absorber units (slurry and scale samples), but B-U1_reuse_ is a notable exception to this conclusion. Even though B-U1_reuse_ had a similar chemical composition to source and makeup waters, bacterial communities associated with this system exhibited low Chao1 and Shannon diversity indices in a fashion similar to the slurries and scales (Table 3).

**Figure 3:**
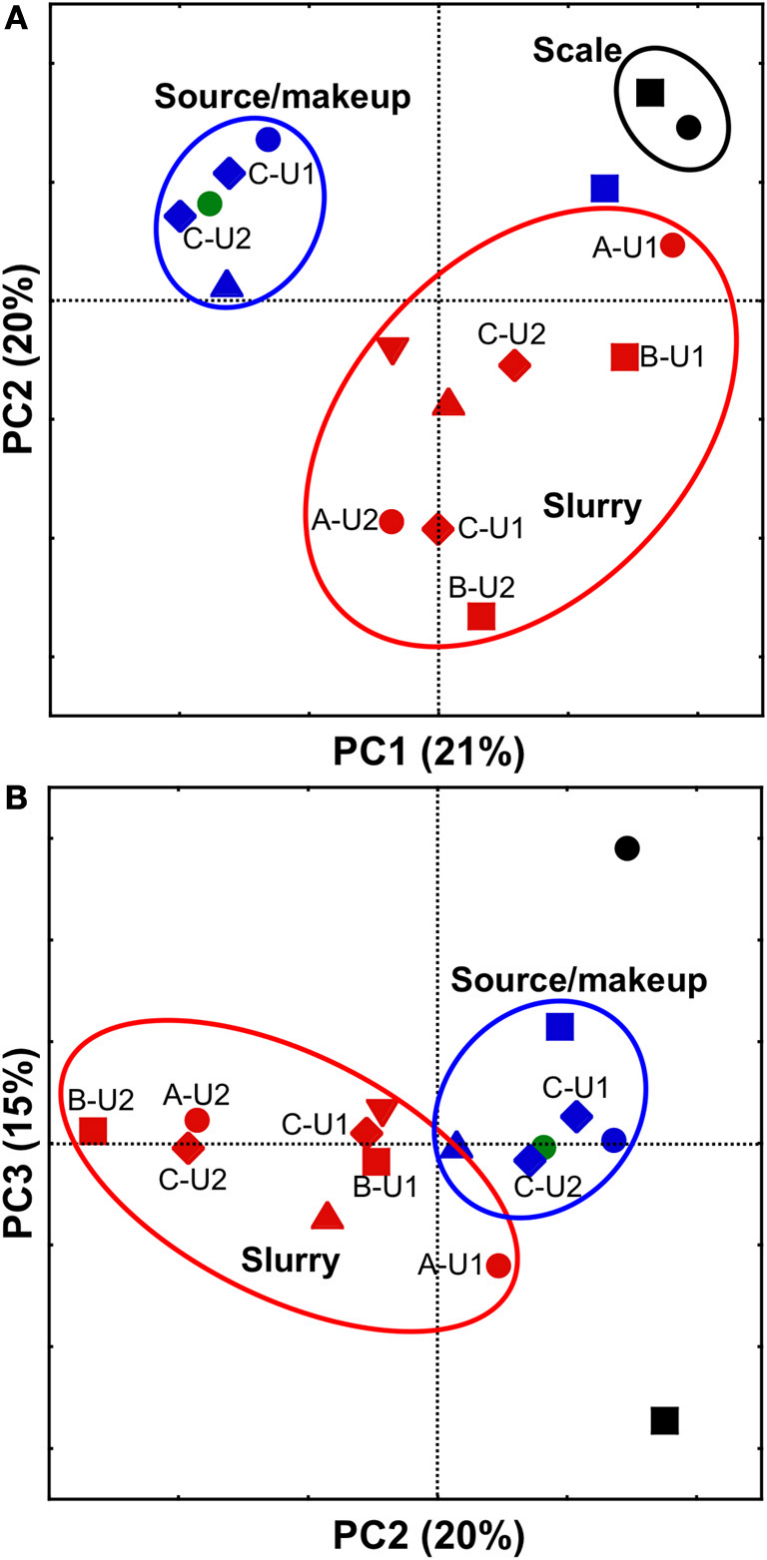
**PCoA analysis of scale- (black shapes), slurry- (red shapes), makeup water- (blue shapes), and source water- (green shape) associated bacterial communities using weighted UniFrac (Lozupone and Knight, 2005).** Scatterplot of PC1 vs. PC2 is shown in **(**panel **A)**, and scatterplot of PC2 vs. PC3 is shown in **(**panel **B)**. Microbial communities observed in facilities (A, B, C, D, and E) are depicted using •, ■, ♦, ▲, and ▼, respectively. In cases where more than one unit from the same facility was evaluated, unit numbers are shown next to their respective shape. Ovals are used to aid in visualization of “clustering” of microbial communities.

**Figure 4:**
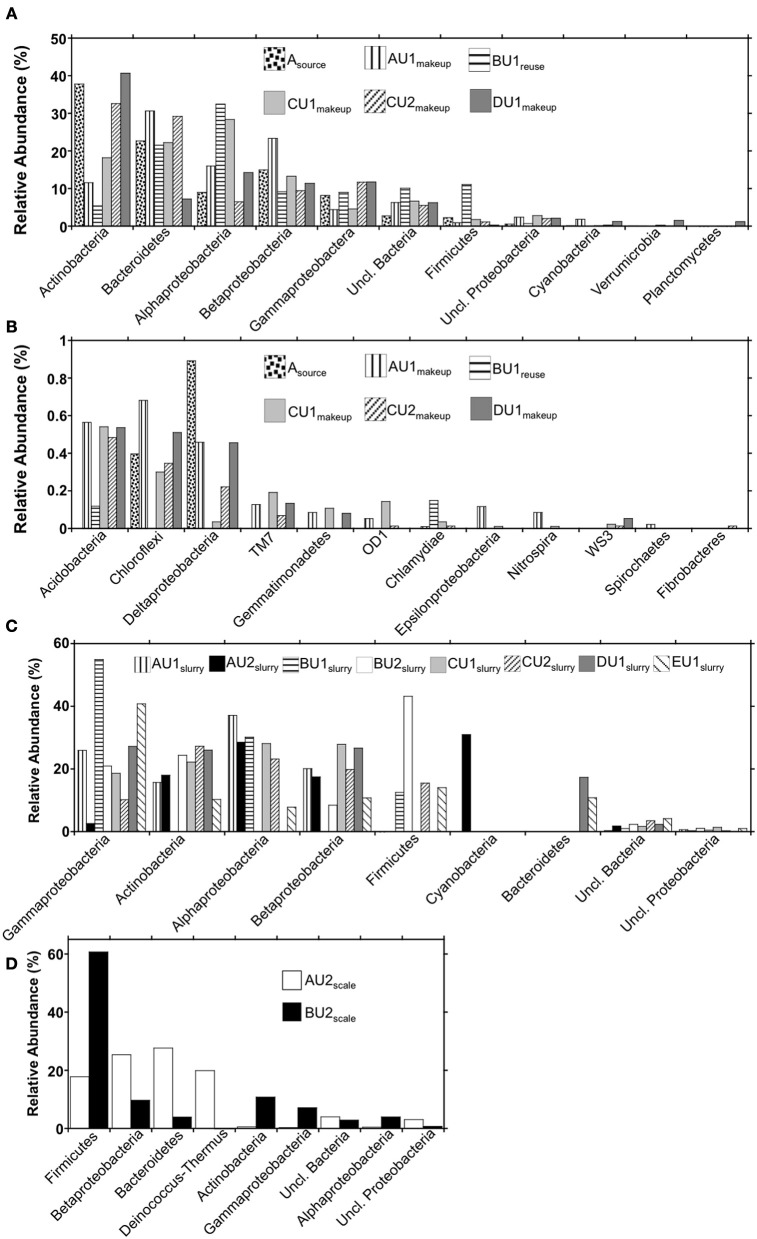
**Phylum-level (and class-level in the cases of the Proteobacteria) bacterial 16S rRNA gene OTU abundances in (panels A and B) makeup, reuse, and source waters, (panel C) absorber slurries, and (panel D) wall scales recovered from FGD units**.

All FGD-associated microbial communities were compared using PCoA with the weighted UniFrac metric, which allows the simultaneous comparison of microbial communities present in several environments by evaluating the common evolutionary history of organisms present in those systems (Lozupone and Knight, 2005). Comparison of FGD-associated microbial communities visualized by plotting PC1 vs. PC2 revealed distinct clustering of “fresh” waters, slurries, and scales (Figure 3A). This clustering pattern may be attributable to the physicochemical conditions to which the microbial communities were exposed. The “fresh” waters were exposed to ambient air temperatures and contained low concentrations of dissolved solutes (Table 1). Slurry-associated microbial communities were exposed to higher concentrations of dissolved solutes (Table 1), and variably high and low temperatures as the slurries were circulated through the FGD absorber units (Figure 1). Scale-associated microorganisms were exposed to continuously high temperatures and solute concentrations, since they were not circulated through the units. Notably, the B-U1_reuse_ community did not cluster with the makeup or source waters in the PC1 vs. PC2 plot, even though it was exposed to physicochemical conditions similar to those of the other “fresh” waters (Table 1). As was observed with the culture-dependent enumerations and diversity metrics, this appears to be a reflection of the fact that B-U1_reuse_ is derived from treated absorber slurry. Indeed, the B-U1_reuse_ community appeared to cluster with the slurry-associated communities in the plot of PC1 vs. PC2 (Figure 3A). Clustering of communities based on FGD unit sampling location (and associated differences in physicochemical conditions) was not as pronounced in the PC2 vs. PC3 plot (Figure 3B). Separation of slurries and source/makeup waters was not observed by PC3 (Figure 3B). It is notable that PC3 allowed clustering of B-U1_reuse_ with the source/makeup waters, which had similar physicochemical conditions (Figure 4B). The scale-associated communities remained well-separated from those of the slurries and source/makeup/reuse microbial communities, though they were separated by PC3 into different quadrants (Figure 3B).

These results suggest that the variability in physicochemical environment to which the microbial communities are exposed leads to the development of microbial communities with differing structures. For instance, exposure of slurry-associated microbial communities to higher solute concentrations and periodically higher temperature induces structural shifts from the “fresh” water microbial communities. However, since the slurry is continually replenished with makeup or reuse water, the slurries retain some characteristics of the “fresh” waters. Microbial communities appear to develop in scales that are quite distinct from either the slurries or the makeup/source waters, since they are continuously exposed to higher temperatures and higher dissolved solute concentrations.

### Evaluation of taxa in “fresh” waters and absorber slurries

Using the RDP classifier function (Wang et al., 2007) in the MacQIIME environment (Caporaso et al., 2010b), we were able to resolve 90–97%, 96–99.4%, and 96–97% of sequences to the phylum-level in “fresh” waters, slurries, and scales, respectively (Figures 4A,C,D). RDP classifier was able to assign as few as 41% of OTUs and as many as 86% of OTUs to the genus level in slurries and “fresh” waters (Table 3). The majority of OTUs detected in the “fresh” waters were affiliated with Actinobacteria, Bacteroidetes, Alphaproteobacteria, Betaproteobacteria, Gammaproteobacteria, and Firmicutes (Figure 4A), though 15 other phyla were minimally abundant (i.e., ≤1%) in at least one of the “fresh” water settings (Figure 4B). These patterns of phylum-level distribution are similar to other freshwater systems (Burkert et al., 2003; Allgaier and Grossart, 2006; Newton et al., 2007; Mueller-Spitz et al., 2009), including those that were characterized using similar sequencing effort (Clingenpeel et al., 2011). It is notable that the B-U1_reuse_ had fewer phyla represented (7) than in makeup waters (15–19), and this is reflected in the diversity indices determined for B-U1_reuse_ (Table 3).

Microbial communities associated with slurries exhibited phylum-level profiles similar to those of the source and makeup waters, with abundant Actinobacteria-, Gammaproteobacteria-, Betaproteobacteria-, and Alphaproteobacteria-affiliated OTUs (Figures 4A,B). However, the minimally abundant phylotypes (Figure 4B) detected in the “fresh” waters were not detected in the slurries, despite similar sampling effort (Figure 2). Indeed, only four (B-U1_slurry_, C-U1_slurry_) or five (A-U1_slurry_, A-U2_slurry_, B-U2_slurry_, C-U2_slurry_, D-U1_slurry_) unique phyla were detected in absorber slurries (Figure 4C). Bacteroidetes-affiliated OTUs, which comprised ≥10% of OTUs detected in “fresh” waters, were only detected in D-U1_slurry_ and E-U1_slurry_ (Figure 1). Firmicutes, which generally comprised a relatively small fraction of the total OTUs in “fresh” waters, were enriched in B-U1_slurry_, B-U2_slurry_, C-U2_slurry_, and E-U1_slurry_ (Figures 4A,C).

Genus-level assignments that could be made to OTUs detected in absorber slurries were examined in more detail and are presented in Table A1. While many of the genera that were abundant in absorber slurries contained halophilic and/or thermophilic representatives (Table A1), these genera are also routinely detected in less “extreme” settings, including soils, freshwater, and marine aquatic settings, and associated with plant and animal hosts. *Salinicoccus*- and *Jeotgalicoccus*-affiliated phylotypes were detected in B-U1_slurry_ and C-U2_slurry_, respectively, and these are the only genera that are composed exclusively of halophilic species, though these genera are poorly represented in culture (Yoon et al., 2003; Hoyles et al., 2004; Claus et al., 2006; Guo et al., 2010; Liu et al., 2011; Martin et al., 2011). The majority of genera detected in the absorber slurries were aerobic or facultatively anaerobic organotrophic bacteria (Table A1), except for *Limnobacter*- (A-U1_slurry_ and A-U2_slurry_) and Cyanobacteria Group VIII-affiliated (A-U2_slurry_) phylotypes (Table A1). *Limnobacter* spp. are aerobic sulfur-oxidizing bacteria and may metabolize partially oxidized sulfur species in the absorber unit (Spring et al., 2001; Lu et al., 2010). The abundance of Cyanobacteria-affiliated OTUs in A-U2_slurry_ was surprising since slurries are not exposed to sunlight, but this unit had started operation ~1 month before samples were collected, so their presence may be attributable to the source water used to fill the absorber unit. Given the cosmopolitan genera associated with the absorber slurries, the slurry-associated microbial communities appear to be a partial reflection of the “fresh” water-associated microbial communities. The high temperature and solute concentrations of the absorber units induce shifts in the microbial communities, but these shifts may be minimized by the continuous cycling of fluids into and out of the absorber unit as well as the continuous replenishment of “fresh” water.

### Evaluation of taxa in scales

While there appeared to be similarities between the “fresh” water- and absorber slurry-associated microbial communities, UniFrac-based evaluation of the microbial communities indicated that scale-associated microbial communities were dramatically different from “fresh” waters or absorber slurries (Figure 3). These differences were also evident in the genera present in the scales. Genus-level assignments could only be made for ~20% of the sequences recovered from the scale samples (Table 3), and in several cases, the numerically dominant OTUs within a phylum could not be resolved beyond the phylum-level using the RDP classifier function (e.g., Betaproteobacteria in A-U2_scale_ and Firmicutes in B-U2_scale_). As such, unresolvable OTUs were evaluated in more detail using BLASTN (Altschul et al., 1990) to compare these OTUs to sequences in the GenBank database. While phyla associated with “fresh” waters and absorber slurries were also detected in scales, more detailed evaluation of 16S rRNA gene sequences recovered from A-U2_scale_ and B-U2_scale_ revealed that the majority (80 and 60%, respectively) of the OTUs were attributable to thermophilic lineages.

Four phyla (Bacteroidetes, Betaproteobacteria, Deinococcus-Thermus, and Firmicutes) comprised 90% of the OTUs detected in A-U2_scale_ (Figure 4D). Deinococcus-Thermus-affiliated phylotypes were not detected in any of the “fresh” water or absorber slurries. All Deinococcus-Thermus-affiliated OTUs were *Meiothermus*- (99% similar to *Meiothermus timidus*; Pires et al., 2005) and *Thermus*- (100% similar to *Thermus scotoductus*; Gounder et al., 2011) affiliated phylotypes. Both of these species are thermophilic aerobic organotrophs, and *T. scotoductus* is capable of anaerobic respiration using Fe(III) and S^0^ as a terminal electron acceptors (Kieft et al., 1999; Pires et al., 2005). While Bacteroidetes-, Betaproteobacteria-, and Firmicutes-affiliated bacteria were detected in “fresh” water and absorber slurries, the predominant OTUs affiliated with these lineages were quite different from those detected in the “fresh” waters or absorber slurries. The most abundant Bacteroidetes-affiliated OTU in A-U2_scale_ was 99% similar to an organism isolated from geothermal soil (Stott et al., 2008). The most abundant Betaproteobacterial OTU in A-U2_scale_ was 92% similar to *Hydrogenophilus thermoluteolus*, a thermophilic H_2_-oxidizing bacterium (Hayashi et al., 1999). The most abundant Firmicutes-affiliated OTU from A-U2_scale_ was 92% similar to 16S rRNA gene sequences recovered from composting operations in the thermophilic phase of operation (Partanen et al., 2010).

Firmicutes-affiliated OTUs were most abundant (61%) in B-U2_scale_, with lower abundances of Actinobacteria, Alphaproteobacteria, Betaproteobacteria, Gammaproteobacteria, Bacteroidetes, and Deinococcus-Thermus (Figure 4D). Approximately 58% of the total OTUs in B-U2_scale_ were 95% similar to *Alicyclobacillus pohliae*, an aerobic, thermophilic Firmicutes isolated from geothermal soils (Imperio et al., 2008). Other thermophilic lineages that were detected in B-U2_scale_ were *Rubrobacter* sp. (Stackebrandt and Schumann, 2006), *Meiothermus* sp. (da Costa et al., 2006), and *Thermithiobacillus* (Kelly and Wood, 2000), though these phylotypes were present in considerably lower abundances than the *Alicyclobacillus*-affiliated phylotypes. The remaining phylotypes detected in B-U2_scale_ were generally cosmopolitan, mesophilic lineages, including phylotypes attributable to *Flavobacterium*, *Hymenobacter*, and *Mucilaginobacter* (Bacteroidetes), *Corynebacterium* and *Arthrobacter* (Actinobacteria), *Pelomonas* (Betaproteobacteria), *Stenotrophomonas* and *Pseudomonas* (Gammaproteobacteria), and *Rhodospirillum* and *Novosphingobium* (Alphaproteobacteria), which were also detected in “fresh” waters and absorber slurries.

## Discussion

### Implications for wet FGD system operation and performance

We observed three microbial community types associated with limestone forced air oxidation wet FGD systems that appeared to be controlled by the prevailing physicochemical conditions specific to the three process points evaluated in the FGD systems. Microbial communities associated with source and makeup waters were numerically dominated by phylotypes associated with mesophilic lineages of Actinobacteria, Bacteroidetes, Firmicutes, Alphaproteobacteria, Betaproteobacteria, and Gammaproteobacteria (Figure 4A), with similar phylum-level profiles to freshwater lakes and rivers (Burkert et al., 2003; Allgaier and Grossart, 2006; Newton et al., 2007; Mueller-Spitz et al., 2009; Clingenpeel et al., 2011). Absorber slurry-associated microbial communities, which were exposed to higher dissolved solid concentrations and periodically higher temperatures, shared some characteristics with “fresh” FGD process waters, but were considerably less diverse. Absorber slurry microbial communities were numerically dominated by Actinobacteria-, Alphaproteobacteria-, Betaproteobacteria-, and Gammaproteobacteria-affiliated phylotypes, though the minimally abundant phylotypes that were detected in the “fresh” FGD process waters were not detected in absorber slurries, despite robust sampling effort. These results suggest that absorber slurry-associated microbial communities retain characteristics of “fresh” FGD process waters, but that the more extreme physicochemical conditions inside the absorber unit induce shifts in the microbial communities. Indeed, the finding of higher numbers of culturable halothermophilic/tolerant bacteria in absorber slurries than in “fresh” FGD process waters supports the hypothesis that “fresh” FGD process water-associated microbial communities adapt to the higher solute concentrations and temperatures of the FGD absorber units. The slurry-associated microbial community structure appears to be retained when slurry waters are treated and returned to circulation as reuse water. Despite the chemical similarities of B-U1_reuse_ to other “fresh” process waters, the B-U1_reuse_-associated microbial communities exhibited diversity and UniFrac characteristics that were more similar to those of absorber slurry-associated communities, and retained relatively high numbers of culturable halothermophilic/tolerant microorganisms.

While absorber slurry-associated microbial communities differ from “fresh” water-associated communities, the retention of some phylotypes is attributable to the dynamics of absorber operation. Absorber units are initially filled with fresh waters, and during unit operation, the slurries are continuously circulated through the system (Figure 1). Slurry residence times in absorber units are typically 10–20 h (Sargent and Lundy, 2003), though this may vary depending on absorber unit size and design. Heat from the flue gas maintains temperatures of 50–80°C in the absorber unit, while fluids outside the unit (i.e., circulating slurry, freshly prepared limestone-water slurry, and source water) are subjected to ambient air temperatures (yearly averages of 10–15°C in the Midwestern United States). When slurries circulate out of the absorber units, they return to ambient temperature while a portion of slurry is diverted for gypsum recovery and water treatment. Slurry that is to be returned to the absorber unit is then amended with fresh limestone and “topped-off” with makeup or reuse water to account for fluid lost to gypsum recovery and evaporation (Figure 1). As such, slurry-associated microbial communities are not continuously exposed to high temperature, and are replenished with organisms associated with “fresh” FGD process waters.

Surprisingly few of the phylotypes detected in the FGD absorber slurries were affiliated with sulfur-metabolizing lineages, despite the abundance of sulfate (and likely partially reduced S species) and the application of robust sampling effort. The only phylotype detected in absorber slurries that was affiliated with an S-metabolizing bacterial lineage was attributable to S-oxidizing *Limnobacter* spp. in A-U1_slurry_ (Table A1). Low numbers of phylotypes attributable to reduced S-oxidizing *Thiobacillus* spp., *Thiothrix* spp. (Lane et al., 1992), and *Rhodopseudomonas* spp. (Then and Trüper, 1981) were detected in “fresh” FGD process waters, but not in slurries. Similarly, phylotypes attributable to Firmicutes- and Deltaproteobacteria-affiliated sulfate-reducing bacteria (Muyzer and Stams, 2008) were detected in low numbers in “fresh” waters, but not at all in absorber slurries or scales. In thermal settings, S metabolism may be mediated by Archaea (Amend and Shock, 2001), which were not evaluated in this study, but the majority of phylotypes detected in the slurries were affiliated with aerobic organotrophic lineages (Table A1). Given the abundant organic carbon supplied by flue gas and abundant O_2_ supplied by forced air, it appears that aerobic organotrophy is the major mode of bacterial metabolism in the FGD slurries.

Scale-associated microbial communities were quite distinct from those of “fresh” FGD process waters and absorber slurries, with abundant phylotypes associated with thermophilic bacterial lineages. Microorganisms associated with absorber slurries are periodically exposed to temperatures of 50–80°C, but the duration of exposure to high temperature is apparently insufficient to induce shifts to the thermophilic microbial communities that we observed in scales recovered from FGD absorber walls. While microbial communities associated with both A-U2_scale_ and B-U2_scale_ were numerically dominated by thermophilic phylotypes, there were differences between the communities. A-U2_scale_ included 16S rRNA gene sequences related to *Hydrogenophilus*, *Thermus*, and *Meiothermus* species as numerically dominant phylotypes, while the majority of 16S rRNA gene sequences detected in B-U2_scale_ were attributable to the genus *Alicyclobacillus*.

The source of the thermophilic bacteria detected in the scales is unclear. Endolithic thermophiles entrained in the limestone feed (Amend and Shock, 2001; Horath and Bachofen, 2009) may have served as an inoculum for the scales, though we were unable to obtain limestone samples from the power generation facilities. It is notable that the thermophilic lineages detected in the scales were not detected in the “fresh” waters, or even in the slurries. The detection of mesophilic phylotypes in scales suggests some influence of the “fresh” process water microbial communities on scale-associated community composition, and it is possible that thermophiles were present in “fresh” waters, but were not detected in due to the application of insufficient sampling effort to “fresh” waters. Thermophilic bacteria have been isolated from temperate soils, and may maintain some level of metabolic activity at relatively low temperatures (i.e., ≤25°C; Marchant et al., 2002, 2008; Hubert et al., 2010; Portillo et al., 2012). These findings suggest that some thermophilic microorganisms may be cosmopolitan components of non-thermal ecosystems (albeit in low abundance), as members of the “rare biosphere” (Sogin et al., 2006; Elshahed et al., 2008; Galand et al., 2009). Indeed, *Thermus*-affiliated species were isolated from hot-water heaters that received water from municipal wells (Brock and Boylen, 1973), suggesting that non-thermal source waters may provide an inoculum for the thermophilic scale-associated communities. While halothermophilic/tolerant organisms were not detected in A_source_, they were detected in low abundances in several of the makeup and reuse water samples (Table 3).

The corrosion of steel structures associated with FGD systems is an increasingly prevalent problem (Mansfield and Jeanjaquet, 1987; Hibner and Ross, 1988; Bordziłowski and Darowicki, 1998; Kim et al., 2010). The high solute concentrations and DO of the absorber unit fluids makes them aggressively corrosive solutions, and may give rise to severe pitting corrosion, which has been reported for FGD structures (including systems evaluated in this study) and is often associated with MIC (Franklin et al., 1991; Strehblow, 2007; Moskal, 2011). The FGD units at Facility A have not yet experienced serious corrosion problems, but Facility B units have experienced severe problems with corrosion, despite the fact that both units were constructed using the same type of steel. The potential for MIC in FGD systems has been recognized, though studied in minimal detail (Moskal, 2011), and it is difficult to definitively determine the contribution of MIC to the deterioration of the absorber units at Facility B or industry-wide. Nevertheless, any MIC occurring in FGD systems is likely to be driven primarily by the distinct thermophilic microbial communities that develop on steel surfaces in the absorber unit wall scale.

Both A-U2_scale_ and B-U2_scale_ contained abundant phylotypes attributable to thermophilic bacterial lineages, but the metabolism of organisms associated with the lineages are quite different. Cultured representatives of the genera *Meiothermus* and *Thermus* are obligately aerobic organotrophs (Loginova et al., 1984; Tenreiro et al., 1995; Chung et al., 1997; Chen et al., 2002; Pires et al., 2005; da Costa et al., 2006), though *Thermus scotoductus* is a facultative anaerobe capable of oxidation of organic carbon or H_2_ with Fe(III) reduction (Kieft et al., 1999), and cultured representatives of the genus *Hydrogenophilus* are aerobic H_2_-oxidizing thermophiles (Hayashi et al., 1999). Hydrogenotrophic consumption of cathodically produced H_2_ is a major mechanism of steel corrosion under anaerobic conditions (Kielemoes et al., 2000; Jack, 2002; Uchiyama et al., 2010; Davidova et al., 2012), but the role of this process under aerobic conditions is unclear, and it appears unlikely to be occurring to any substantial extent in A-U2_scale_, since no corrosion has been reported at this facility thus far. *Alicyclobacillus* species, such as those detected in B-U2_scale_, metabolize carbohydrates to organic acids, and several species oxidize Fe(II) and reduced sulfur species (Dufresne et al., 1996; Jiang et al., 2008; Imperio et al., 2008; Guo et al., 2009). The abundance of phylotypes attributable to *Alicyclobacillus* in B-U2_scale_ is quite striking, and both organotrophic (organic acid production) and lithotrophic (Fe(II) oxidation) activities are substantial contributors to steel corrosion (Rao et al., 2000; Starosvetsky et al., 2001; Pecar et al., 2011). Indeed, slightly higher Fe(III) concentrations were detected in B-U2_scale_ than in A-U2_scale_ (Table 2), which may indicate Fe(II)-oxidizing activities.

Our results suggest that unique microbial communities develop in physicochemically distinct process points within FGD systems. Microbial communities associated with makeup waters have characteristics typical of freshwater bodies. Microbial communities associated with slurries bear some resemblance to the makeup waters, but are influenced by the high dissolved solute concentration of the slurry fluids, giving rise to more abundant halophilic/tolerant microorganisms. Microbial communities associated with scale deposits are quite unique, with abundant phylotypes attributable to thermophilic bacterial lineages. Ultimately, activities of the scale-associated microbial communities will be the predominant drivers of MIC in FGD systems, and the dynamics of their activities will need to be evaluated in more detail to determine their contribution to the corrosion of steel structures within these facilities.

### Conflict of interest statement

The authors declare that the research was conducted in the absence of any commercial or financial relationships that could be construed as a potential conflict of interest.

## Appendix

**Table A1:** **Genus-level taxonomic assignments of abundant (≥20%) phylotypes affiliated with abundant (≥8%) phyla (and classes, in the case of the Proteobacteria) detected in slurries of FGD absorber units. If halophilic or thermophilic representatives of a given genus have been described, this is noted by a (+). Relevant references are provided as superscript numbers and full citations are provided below the table**.

**Slurry sample**	**Phylum**	**Predominant genus**	**Halophilic**	**Thermophilic**	**Comments**
A-U1_slurry_	Alphaproteobacteria (37%)	*Sphingopyxis* (99%)	+^1^	-	Aerobic organoheterotrophs; isolated from soil, activated sludge, and marine systems^1−4^
	Gammaproteobacteria (25%)	*Stenotrophomonas* (97%)	+^5^	-	Common soil and sediment-associated bacteria; also isolated from saline industrial settings^5^
	Betaproteobacteria (20%)	*Limnobacter* (99%)	-	-	Aerobic, thiosulfate oxidizers; isolated from freshwater lakes and fresh volcanic deposits^6, 7^
	Actinobacteria (16%)	*Solirubrobacter* (99%)	-	-	Mesophilic soil bacteria^8, 9^
A-U2_slurry_	Cyanobacteria (31%)	Group VIII^*^ (100%)	+^10, 11^	+^12^	Oxygenic phototrophs
	Alphaproteobacteria (21%)	*Paracoccus* (84%)	+^13-15^	-	Encountered in soil aquatic and industrial settings
	Actinobacteria (18%)	*Arthrobacter* (62%)	+^16-18^	-	Aerobic soil organotrophs^16-18^
		*Propionibacterium* (21%)	-	+^19^	Associated with animals, plants; dairy industrial importance; strains shown to tolerate temperatures as high as 90°C^19^
	Betaproteobacteria (17%)	*Limnobacter* (41%)	-	-	Aerobic, thiosulfate oxidizers; isolated from freshwater lakes and fresh volcanic deposits^6, 7^
		*Ralstonia* (24%)	+^20^	-	Common in soil; found in saline soil^20^
		*Aquabacterium* (20%)	-	-	Isolated from drinking water and freshwater springs^20^
B-U1_slurry_	Gammaproteobacteria (55%)	*Stenotrophomonas* (75%)	+^5, 24^	-	Widely distributed in soil and aquatic settings^5, 24^
		Enterobacteriaceae^*^ (21%)	+^5, 25^	+^5^	Widely distributed, including halophilic and thermophilic representatives in industrial settings^5^
	Alphaproteobacteria (30%)	*Phyllobacterium* (92%)	-	-	Aerobic organotrophic isolates from soil, water, and plants^26^
	Firmicutes (13%)	*Salinicoccus* (99%)	+^27^	-	Halophilic genus^27^
B-U2_slurry_	Firmicutes (43%)	*Streptococcus* (31%)	-	+^28^	Normally plant- or animal-associated; thermophilic/tolerant strains resistant to pasteurization^28, 29^
		*Sporosarcina* (29%)	+^30-34^	+^70^	–
		*Veillonella* (29%)	-	-	Oral bacteria^72^
	Actinobacteria (24%)	*Propionibacterium* (97%)	-	+^19^	Associated with animals, plants; dairy industrial importance; strains shown to tolerate temperatures as high as 90°C^19^
	Gammaproteobacteria (21%)	*Acinetobacter* (35%)	+^35, 36^	+^37^	Widespread in soil, sediment, and aquatic systems; includes halophilic^35, 36^ and thermophilic^37^ representatives
B-U2_slurry_		*Stenotrophomonas* (22%)	+^5, 24^	-	Widely distributed in soil and aquatic settings^5, 24^
	Betaproteobacteria (8%)	*Limnobacter* (55%)	-	-	Aerobic, thiosulfate oxidizers; isolated from freshwater lakes and fresh volcanic deposits^6, 7^
C-U1_slurry_	Alphaproteobacteria (28%)	*Bradyrhizobium* (92%)	+^38^	-	Widespread in soil; one halophilic isolate^38^
	Betaproteobacteria (28%)	*Ralstonia* (97%)	+^20^	-	Common in soil; found in saline soil^20^
	Actinobacteria (22%)	*Solirubrobacter* (53%)	-	-	Mesophilic soil bacteria^8, 9^
		*Marmoricola* (43%)	+^39, 40^	-	All isolated Marmoricola spp. from marine settings or moderately halophilic^39, 40^
	Gammaproteobacteria (19%)	Enterobacteriaceae^*^ (97%)	+^5, 25^	+^5^	Widely distributed, including halophilic and thermophilic representatives in industrial settings^5^
C-U2_slurry_	Actinobacteria (27%)	*Propionibacterium* (69%)	-	+^19^	Associated with animals, plants; dairy industrial importance; strains shown to tolerate temperatures as high as 90°C^19^
		*Corynebacterium* (27%)	+^16, 41^	-	Associated with plants, animals, soils, sediments in fresh and saline settings
	Alphaproteobacteria (23%)	Acetobacteraceae^*^ (98%)	+^42^	-^43^	Genus-level assignment not possible; OTUs 98–99% similar to sequences detected in non-saline soil^44-47^
	Betaproteobacteria (20%)	*Aquabacterium* (92%)	-	-	Isolated from drinking water and freshwater springs^20^
	Firmicutes (16%)	*Jeotgalicoccus* (74%)	+^48-52^	-	All *Jeotgalicoccus* sp. described are halophilic^48-52^
	Gammaproteobacteria (10%)	Enterobacteriaceae^*^ (64%)	+^5, 25^	+^5^	Widely distributed, including halophilic and thermophilic representatives in industrial settings^5^
		Xanthomonadaceae^*^ (35%)	+^53^	+^54, 55^	Widespread in soil and aquatic settings
D-U1_slurry_	Gammaproteobacteria (27%)	*Pseudomonas* (60%)	+^16^	+^56^	Widespread in soil, sediment and aquatic settings
		Enterobacteriaceae^*^ (39%)	+^5^	+^5^	Widely distributed, including halophilic and thermophilic representatives in industrial settings^5^
	Betaproteobacteria (27%)	Commamonadaceae^*^ (98%)	-	-	Genus-level assignment not possible; OTUs 100% similar to *Polaromonas* spp. isolated from water treatment facilities and glacier meltwater^57, 58^
	Actinobacteria (26%)	*Propionibacterium* (80%)	-	+^19^	Associated with animals, plants; dairy industrial importance; strains shown to tolerate temperatures as high as 90°C^19^
	Bacteroidetes (17%)	*Flavobacterium* (100%)	+^16^	-	Aerobic organotrophic bacteria common to freshwater and soil^65^
E-U1_slurry_	Gammaproteobacteria (40%)	Enterobacteriaceae^*^ (81%)	+^5^	+^5^	Widely distributed, including halophilic and thermophilic representatives in industrial settings^5^
		*Pseudomonas* (10%)	+^16^	+^56^	Widespread in soil, sediment and aquatic settings
	Firmicutes (14%)	*Clostridium* (53%)	+^59-61^	+^62-64^	Organotrophic anaerobic bacteria
		*Streptococcus* (41%)	-	+^28^	Normally plant- or animal-associated; thermophilic/tolerant strains resistant to pasteurization^28, 29^
E-U1_slurry_	Bacteroidetes (11%)	*Flavobacterium* (100%)	+^16^	-	Aerobic organotrophic bacteria common to freshwater and soil^65^
	Betaproteobacteria (11%)	*Ralstonia* (98%)	+^20^	-	Common in soil; found in saline soil^20^
	Actinobacteria (10%)	*Propionibacterium* (40%)	-	+^19^	Associated with animals, plants; dairy industrial importance; strains shown to tolerate temperatures as high as 90°C^19^
		*Dietzia* (25%)	+^66^	-	Isolates recovered from soil and freshwater settings^71^
	Alphaproteobacteria (8%)	*Roseomonas* (94%)	+^67^	+^68^	Aerobic isolates from soil, sediments, industrial, and freshwater settings; plant and animal hosts^69^

* Genus-level taxonomic assignments could not be made using the Ribosomal Database Project II Classifier function^73^ in the MacQIIME environment^74^.
